# Intrinsic capacity and recent falls in adults 80 years and older living in the community: results from the ilSIRENTE Study

**DOI:** 10.1007/s40520-024-02822-7

**Published:** 2024-08-10

**Authors:** Stefano Cacciatore, Emanuele Marzetti, Riccardo Calvani, Anna Picca, Sara Salini, Andrea Russo, Matteo Tosato, Francesco Landi

**Affiliations:** 1 Department of Geriatrics, Orthopedics and Rheumatology, Università Cattolica del Sacro Cuore, L.go F. Vito 1, Rome, 00168 Italy; 2 Fondazione Policlinico Universitario “Agostino Gemelli” IRCCS, L.go A. Gemelli 8, Rome, 00168 Italy; 3 Department of Medicine and Surgery, LUM University, SS100 km 18, Casamassima, 70100 Italy

**Keywords:** Intrinsic capacity, Falls, Functional reserve, ADL, Disability, Frailty, Hip fracture, Cognitive decline, Dementia, Malnutrition

## Abstract

**Background:**

Falls in older adults significantly impact overall health and healthcare costs. Intrinsic capacity (IC) reflects functional reserve and is an indicator of healthy aging.

**Aims:**

To explore the association between IC and recent falls (≤ 90 days) in community-dwelling octogenarians from the Aging and Longevity in the Sirente geographic area (IlSIRENTE) study.

**Methods:**

The Minimum Data Set for Home Care (MDS−HC) and supplementary questionnaires and tests were used to assess the five IC domains: locomotion, cognition, vitality, psychology, and sensory. Scores in each domain were rescaled using the percent of maximum possible score method and averaged to obtain an overall IC score (range 0−100).

**Results:**

The study included 319 participants (mean age 85.5 ± 4.8 years, 67.1% women). Mean IC score was 80.5 ± 14.2. The optimal IC score cut-off for predicting the two-year risk of incident loss of at least one activity of daily living (ADL) was determined and validated in a subset of 240 individuals without ADL disability at baseline (mean age 84.7 ± 4.4 years, 67.1% women). Participants were then stratified into low (< 77.6) and high (≥ 77.6) IC categories. Those with high IC (63.9%) were younger, more often males, and had lower prevalence of recent falls, disability, multimorbidity, and polypharmacy. Logistic regression models including IC as a continuous variable revealed a significant association between higher IC and lower odds of falls. This association was significant in the unadjusted (odds ratio [OR] 0.96, 95% confidence interval [CI] 0.94–0.98, *p* < 0.001), age- and sex-adjusted (OR 0.96, 95% CI 0.94–0.98, *p* < 0.001), and fully adjusted models (OR 0.96, 95% CI 0.93–0.99, *p* = 0.003). When considering IC as a categorical variable, unadjusted logistic regression showed a strong association between high IC and lower odds of falls (OR 0.31, 95% CI 0.16–0.60, *p* < 0.001). This association remained significant in both the age- and sex-adjusted (OR 0.30, 95% CI 0.15–0.59, *p* < 0.001) and fully adjusted models (OR 0.33, 95% CI 0.16–0.82, *p* = 0.007). The locomotion domain was independently associated with falls in the unadjusted (OR 0.98, 95% CI 0.97–0.99, *p* < 0.001), age- and sex-adjusted (OR 0.97, 95% CI 0.96–0.99, *p* < 0.001), and fully adjusted model (OR 0.98, 95% CI 0.96–0.99, *p* < 0.001).

**Discussion:**

This is the first study using an MDS−HC-derived instrument to assess IC. Individuals with higher IC were less likely to report recent falls, with locomotion being an independently associated domain.

**Conclusions:**

Lower IC is linked to increased odds of falls. Interventions to maintain and improve IC, especially the locomotion domain, may reduce fall risk in community-dwelling octogenarians.

**Supplementary Information:**

The online version contains supplementary material available at 10.1007/s40520-024-02822-7.

## Introduction

Falls are defined as unanticipated incidents in which persons come to rest on the ground, floor, or a lower level, not being caused by abrupt medical illnesses (e.g., seizures, stroke) or external circumstances (e.g., being struck by a moving item) [1]. While falls may occur at any stage of life, they are among the most prevalent and severe events leading to mortality, disability, and increased healthcare costs in older adults [2, 3]. A recent meta-analysis estimated the global prevalence of falls in older adults as high as 26.5% [4]. A previous fall event significantly increases the risk of subsequent falls, particularly in those who are more vulnerable (33–50%) [5, 6]. Additionally, after experiencing a fall, a significant proportion of older adults (21–39%) report the emergence of a newfound fear of falling [7]. Up to one third of falls may result in moderate-to-severe injuries, such as hip fractures or brain injuries, requiring either hospitalization or at least one day of restricted activity [8, 9]. Falls that need hospitalization are associated with significant mortality rates both in the short and the long term, with 17.2% of fallers dying within a month and up to 50% dying within a year [10]. The financial consequences of fall-related injuries in older adults in the United States account for nearly $20 billion in yearly expenses for immediate medical care and an estimated $50 billion in total annual healthcare expenditures [11–13].

The World Health Organization (WHO) has defined healthy aging as the process of achieving and sustaining functional ability, determined by the interactions between environmental factors and the intrinsic capacity (IC) of an individual across the lifespan [14, 15]. By shifting the perspective from cross-sectional evaluations to longitudinal trajectories, this framework approaches healthy aging as an accumulation of functional reserves instead of deficits, setting aside these reserves for the challenges of later life [14–16]. IC is the cornerstone of this model and encompasses functional reserves into the essential domains of locomotion capacity, cognitive abilities, psychological reserves, vital energy, and sensory skills [17].

Despite the growing interest in the concept of IC, there is still no universal agreement on its definition in clinical investigations and its translation into clinical practice [18, 19]. Moreover, while previous studies have established a link between the frailty status and a higher risk of falling in older adults [20], evidence on the association between decreased IC and risk of falls is sparse. Therefore, the aim of this investigation was to explore the association between IC and recent falls in a cohort of Italian community-dwelling octogenarians enrolled in the “Aging and Longevity in the Sirente geographic area” (ilSIRENTE) study.

## Methods

The ilSIRENTE was a prospective cohort study conducted among the population living in the Sirente geographic area (L’Aquila, Abruzzo, Italy). The study was designed by the Department of Geriatrics of the Università Cattolica del Sacro Cuore (Rome, Italy) and was conducted in partnership with local authorities and primary healthcare practitioners affiliated with the municipalities of the Sirente Mountain Community. The study protocol is thoroughly described elsewhere [21]. The research was conducted in adherence to the ethical principles outlined in the Declaration of Helsinki for medical investigations. The Ethics Committee of the Università Cattolica del Sacro Cuore granted approval for the study. A written informed consent was obtained from all participants or their proxies, when appropriate, prior to enrolment.

### Study sample

The study sample consisted of individuals who were born in the Sirente area before 1 January 1924 and were in the same geographical region during the initial survey period. Among the 429 persons who were eligible for inclusion, 65 opted not to participate, leading to a final sample of 364 individuals. For the present study, analyses were performed in a total of 319 participants, after removing individuals with missing data on nutritional status (*n* = 35), cognition (*n* = 15), physical performance (*n* = 14), depression (*n* = 6), fall history (*n* = 3), or vision (*n* = 1). In several instances, participants had missing data in more than one domain, which explains why the sum of individuals with unavailable data exceeds the number of excluded participants.

### Data collection

Baseline assessments of participants started in December 2003 and were completed in September 2004. The Minimum Data Set for Home Care (MDS−HC) instrument was used to collect data on study participants, in accordance with the procedures detailed in the MDS−HC manual [22]. The MDS−HC comprises more than 350 domains, which include sociodemographics, assessments of physical and cognitive status, significant clinical diagnoses, and a wide range of signs, symptoms, syndromes, and medications [22]. Supplementary information pertaining to lifestyle habits, levels of physical activity, and physical performance was collected using questionnaires and tests employed in the “Invecchiare in Chianti Study” (InCHIANTI) study [23]. Excessive alcohol consumption was operationalized as a daily drinking of above 500 mL of wine (or an equivalent amount of alcohol). Current smoking was defined as the habitual consumption of tobacco, occurring at a frequency of at least once per week, throughout the preceding year. Clinical diagnoses were documented by the study physicians through the collection of information from the participant and their primary care physician, doing a physical examination, and conducting a thorough evaluation of clinical documentation including laboratory testing and imaging examinations. As reported in previous studies [24], the participant’s level of physical activity was evaluated based on their engagement in activities requiring energy expenditure, including leisure and work-related activities. Participants were defined physically active if they reported engaging in moderate-intensity activities for a minimum of two hours per week during the previous year. Multimorbidity was operationalized as the simultaneous presence of two or more chronic diseases, while polypharmacy was defined as the habitual use of at least five medications. Body mass index (BMI) was determined by dividing body mass in kg by the square of height in meters. MDS-derived basic (ADL) and instrumental activities of daily living (IADL) were used to determine disability status in the seven days prior to the assessment [25, 26]. The ADL scale examines self-performance of mobility in bed, transfers, locomotion, dressing, eating, toilet use, and personal hygiene. The IADL scale assesses the capacity to perform tasks that require the use of various abilities (both physical and cognitive) and that take place in different environments (inside and outside the home). Tasks enlisted in the IADL scale include meal preparation, ordinary housekeeping, managing finances, managing medications, and managing transportation. Both ADL and IADL were coded into an 8-category hierarchical scale, with 0 indicating total independence/capacity and 7 corresponding to total dependance/incapacity [25, 26].

### Development of the intrinsic capacity score

In accordance with the recommendations by the WHO, IC was defined as the composite of five domains: locomotion, cognition, vitality, psychological wellbeing, and sensory function [17].

For the present investigation, locomotion was evaluated through the short physical performance battery (SPPB) [27]. The validity, reproducibility, and safety of the SPPB have been demonstrated in numerous studies [27–30]. The SPPB consists of three subtests that assess standing balance, habitual gait speed, and the capacity to rise from a chair. For the balance test, participants were instructed to stand in three progressively difficult positions for 10 s each. These positions included standing with their feet placed side-by-side, in a semi-tandem stance, and in a complete tandem stance. For the gait speed test, participants were requested to walk at their habitual pace down a 4-m pathway, commencing from a stationary stance. The trial was performed twice with the faster (m/s) utilized to compute the score. For the chair-stand subtest, participants were instructed to perform five repetitions of rising from a chair and sitting down as quick as possible, while maintaining their arms folded over their chest. The time to complete the task was recorded and used for score assignment. Each of the three SPPB subtasks were classified into five levels based on predetermined cut-points. A score of 0 indicates an inability to perform the test, while a score of 4 represents the maximum level of performance for the task. Hence, the summary ranges from 0 to 12.

Cognition was explored through the cognitive performance scale (CPS). The CPS is generated from five items of the MDS (level of consciousness, decision making, short-term memory, making self-understood, and eating performance) [31]. The items are integrated into a single, hierarchical cognitive rating scale with seven categories, ranging from 0 (no cognitive impairment) to 6 (very severe cognitive impairment). The CPS allows robust estimates of cognitive status and shows remarkable agreement with the Mini Mental State Examination (MMSE) [31].

Vitality capacity is defined as the physiological factors that contribute to an individual’s IC, and these may include energy balance and metabolism [32]. For the present investigation, vitality was assessed through the Mini Nutritional Assessment − Short Form (MNA−SF). The MNA−SF is a simple and rapid tool that can be used to effectively screen malnutrition in older adults [33]. It was developed by extracting the six items with the highest sensitivity and overall accuracy for predicting malnutrition from the full 18-item MNA, and subsequently validated in multiple cohorts, including community-dwelling, hospitalized, and institutionalized older adults [33, 34]. The score for the MNA−SF is obtained by answering six specific questions that assess key aspects of an individual’s nutritional status. Each question has different scoring options, and the total possible score ranges from 0 to 14 points. A score of 12−14 points indicates normal nutritional status. A score of 8−11 points suggests risk of malnutrition. A score of 0−7 points indicates malnutrition [33].

Psychological wellbeing was assessed using the MDS depression rating scale (MDS−DRS). The MDS−DRS was developed by combining seven items of the MDS (negative statements, persistent anger, expression of unrealistic fears, repetitive health complaints, repetitive anxious complaints, sad, pained, worried facial expression, and crying or tearfulness) [35]. The total possible score ranges from 0 to 14, and a score of 3 or higher may indicate a potential or actual problem with depression. The MDS−DRS was validated against the Hamilton Depression Rating Scale (HDRS), the Cornell Scale for Depression in Dementia (CSDD), and the Calgary Depression Scale (CDS) and can be used as a clinical screening tool for depression [35].

Sensory function was defined as the combination of quality of vision and hearing patterns item from the MDS−HC. Quality of vision is graded on a scale from 0 (adequate, sees fine details, including regular print in newspapers/books) to 4 (severely impaired, no vision or sees only light, colors or shapes, eyes do not appear to follow objects) [36]. Hearing patterns are classified on a scale from 0 (hears adequately normal talk, TV, phone, doorbell) to 3 (highly impaired, absence of useful hearing) [37].

In order to maintain consistency and facilitate comparison between different variables that define the domains of the IC, data were rescaled using the percent of maximum possible score (POMP) method [38]. Therefore, each variable was transformed to a standardized scale ranging from 0 (representing the minimum possible value) to 100 (corresponding to the maximum possible value). The IC summary score was calculated as the sum of each of the five domains, divided by five and ranged from 0 to 100.

### Validation of the intrinsic capacity score

The IC score was validated to predict the risk of two-year incident disability. Participants had home-based examinations at the beginning of the study and after two years. Baseline and follow-up assessments were conducted to evaluate the existence of impairment across several domains of functioning. The occurrence of functional limitations in completing ADLs was defined as the need for assistance in one or more of the following areas: eating, clothing, moving in home and outside home, transferring, bed mobility, personal hygiene, and using the toilet [39, 40]. Only participants who did not report any impairment at the beginning of the study were included in the analyses to examine the association between decreased IC and the development of incident disability.

### Recent falls

Recent falls were evaluated by a general practitioner, nurses, and a geriatrician as part of the MDS−HC assessment. Participants were instructed to document any instances of falling that had occurred in the previous three months. In the MDS−HC manual [41], a fall is defined as a sudden loss of balance resulting in any part of the body above the feet making contact with the floor [24, 42].

### Statistical analysis

Descriptive statistics were utilized to summarize participant characteristics, stratified by sex, in the subset of participants without impairment in ADL at baseline used to develop and validate IC and IC categories in the full sample. Continuous variables are presented as mean values ± standard deviation, while categorical variables are presented as absolute numbers and percentages. Unpaired t-tests and chi-squared (χ^2^) statistics were used to assess differences between IC categories (low vs. high) for continuous and categorical variables, respectively. All tests were two-tailed with statistical significance set as *p* < 0.05. Receiver operating characteristic (ROC) curves were used to assess the ability of IC to predict incident disability at two years and falls, and to identify the optimal IC cutoff value to define low and high IC. The area under the curve (AUC) was calculated to assess the predictive ability of the model. Logistic regression models were built to calculate unadjusted and adjusted odds ratios (ORs) and 95% confidence intervals (CIs) for incident ADL disability according to IC categories, and to explore the relationship between IC and its subdomains and recent falls. To reduce confounding and improve the precision of estimates, models were adjusted for age, sex, and other predictors previously associated with the occurrence of incident disability and falls within 90 days prior to the evaluation. Variables to be included in the logistic regression models were selected according to statistical significance at the univariate analysis and clinical plausibility. To evaluate the effect of an independent variable on a dependent variable through a mediator, mediation analysis was conducted by regressing the dependent variable on the independent variable (Model A), the mediator on the independent variable (Model B), and finally the dependent variable on both the independent variable and the mediator (Model C) to estimate the direct, indirect, and total effects. The covariates included in the logistic regression model exploring the association between IC and incident disability were age at enrolment, female sex, regular physical activity, years of education, alcohol abuse, and number of diseases. The covariates included in the logistic regression model exploring the association between IC and falls were age at enrolment, female sex, living alone, and number of diseases. All analyses were performed using R version 4.2.3 (R Core Team, Vienna, Austria).

## Results

The mean age of the 319 participants was 85.5 ± 4.8 years, and 214 (67.1%) were women. Individuals with a history of falls within 90 days were less likely to be physically active (39.5% vs. 67.0%, *p* = 0.001), had higher ADL (2.09 vs. 0.859, *p* = 0.004), and IADL scores (3.70 vs. 2.56, *p* = 0.007) indicating greater disability. Additionally, a lower proportion had ADL disability at enrollment (53.5% vs. 78.6%, *p* = 0.001). Nutritional status was poorer in the recent fall group, with a lower MNA−SF score (11.8 vs. 12.5, *p* = 0.044). Physical performance, as measured by the SPPB summary score, was significantly lower in the recent falls group (4.72 vs. 7.33, *p* < 0.001). Hearing impairment showed significant differences, with the recent fall group having higher proportions of mild-to-moderate impairment (69.8% vs. 50.4%, *p* = 0.027) and a lower proportion without impairment (27.9% vs. 47.5%, *p* = 0.025). IC total score was notably lower in the recent falls group (72.9 vs. 81.7, *p* < 0.001). Depression was more prevalent in the recent falls group (39.5% vs. 23.6%, *p* = 0.041). Lastly, the number of diseases was higher among those with recent falls (2.56 vs. 2.08, *p* = 0.046) (Supplementary Table 1).

The optimal IC score cutoff for predicting the two-year risk of incident disability was determined and validated in a subset of 240 individuals without disability at baseline assessment. The general characteristics of the validation sample according to sex are shown in Table 1. The mean age was 84.7 ± 4.4 years and 161 (67.1%) were women. Male participants had a higher education level (*p* = 0.014) and reported more frequently excessive alcohol consumption (*p* < 0.001), active smoking (*p* = 0.002), and physical inactivity (*p* = 0.001) than women. Men had greater MNA−SF scores (*p* < 0.001) and showed higher rates of normal nutritional status (*p* = 0.003), while women were more frequently at risk of malnutrition (*p* = 0.002). Additionally, women had lower SPPB scores (*p* < 0.001) and higher MDS−DRS scores (*p* < 0.001). Finally, men showed higher prevalence of chronic obstructive pulmonary disease (*p* = 0.001) and lower rates of both depression (*p* < 0.001) and osteoarthritis (*p* = 0.001). Although women had a greater number of diseases (*p* = 0.023), there were no statistically significant differences in the prevalence of multimorbidity (*p* = 0.595). The mean IC summary score in the validation sample was 85.9 ± 9.6.

**Table 1:** Characteristics of participants without impairment in activities of daily living at baseline (*n* = 240) according to sex

	Women (*n* = 161)	Men (*n* = 79)	Total sample (*n* = 240)	*p*
*Personal characteristics*				
Age, years	84.5 (4.3)	85.3 (4.6)	84.7 (4.4)	0.156
Education, years	5.0 (1.4)	5.8 (2.6)	5.3 (1.9)	0.014
Living alone	70 (43.5%)	23 (29.1%)	93 (38.8%)	0.060
Alcohol abuse	4 (2.5%)	26 (32.9%)	30 (12.5%)	< 0.001
Active smoking	1 (0.6%)	7 (8.9%)	8 (3.3%)	0.002
Physically active	121 (75.2%)	71 (89.9%)	192 (80.0%)	0.001
Recent fall(s)	17 (10.6%)	6 (7.6%)	23 (9.6%)	0.617
Disabled at follow-up	26 (16.1%)	11 (13.9%)	37 (15.4%)	0.654
*Nutritional status and physical performance*				
BMI, kg/m^2^	26.3 (4.7)	26.1 (3.1)	26.2 (4.3)	0.658
MNA−SF total score	12.7 (1.5)	13.5 (1.2)	13.0 (1.5)	< 0.001
Malnutrition (MNA−SF < 8)	1 (0.6%)	1 (1.3%)	2 (0.8%)	0.605
At risk (MNA−SF 8–11)	30 (18.6%)	3 (3.8%)	33 (13.8%)	0.002
Normal (MNA−SF ≥ 12)	130 (80.7%)	75 (94.9%)	205 (85.4%)	0.003
SPPB summary score	7.9 (2.7)	9.3 (2.5)	8.3 (2.7)	< 0.001
*Cognition and mood*				
CPS score	0.34 (0.79)	0.34 (0.90)	0.34 (0.83)	0.999
MDS−DRS score	1.65 (2.24)	0.43 (1.13)	1.25 (2.03)	< 0.001
*Sensory impairment*				
Hearing impairment				
Absent	88 (54.7%)	37 (46.8%)	125 (52.1%)	0.254
Mild/moderate	71 (44.1%)	41 (51.9%)	112 (46.7%)	0.255
Severe	2 (1.2%)	1 (1.3%)	3 (1.3%)	0.974
Vision impairment				
Absent	104 (64.6%)	51 (64.6%)	155 (64.6%)	0.999
Mild/moderate	25 (15.5%)	16 (20.3%)	41 (17.1%)	0.361
Severe	32 (19.9%)	12 (15.2%)	44 (18.3%)	0.378
*Clinical characteristics*				
Coronary artery disease	20 (12.4%)	9 (11.4%)	29 (12.1%)	0.985
Heart failure	7 (4.3%)	2 (2.5%)	9 (3.8%)	0.722
Diabetes mellitus	27 (16.8%)	13 (16.5%)	40 (16.7%)	0.999
COPD	10 (6.2%)	17 (21.5%)	27 (11.3%)	0.001
Dementia	4 (2.5%)	3 (3.8%)	7 (2.9%)	0.687
Parkinson’s disease	1 (0.6%)	1 (1.3%)	2 (0.8%)	0.551
Depression	47 (29.2%)	6 (7.6%)	53 (22.1%)	< 0.001
Cancer	5 (3.1%)	3 (3.8%)	8 (3.3%)	0.721
Osteoarthritis	43 (26.7%)	6 (7.6%)	49 (20.4%)	0.001
Number of diseases	2.1 (1.3)	1.8 (1.1)	2.0 (1.2)	0.023
Multimorbidity	101 (62.7%)	46 (58.2%)	147 (61.3%)	0.595
Number of medications	3.1 (2.1)	2.80 (2.2)	3.0 (2.1)	0.337
Polypharmacy	42 (26.1%)	15 (19.0%)	57 (23.8%)	0.292

Data are reported as means (standard deviations) and absolute numbers (%) for continuous and categorical variables, respectively

Alcohol abuse: ≥500 mL daily of wine or equivalent; multimorbidity: ≥2 chronic diseases; polypharmacy: ≥5 medications

Abbreviations: BMI: body mass index; CPS: cognitive performance scale; COPD: chronic obstructive pulmonary disease; MDS−DRS: Minimum Data Set − Depression Rating Scale; SPPB: short physical performance battery

To evaluate the relationship between IC, physical activity, and incident disability, mediation analysis was performed (Supplementary Table 2). In Model A, the IC effect on incident disability was −0.014 (*p* < 0.001), indicating a significant negative relationship where higher IC is associated with less incident disability. Model B showed that IC positively impacted physical activity, with an effect of 0.045 (*p* = 0.003), suggesting a significant positive association. In Model C, including both IC and physical activity impacting incident disability, the IC effect on incident disability decreased to −0.016 (*p* < 0.001), indicating that the IC remained significant after accounting for physical activity, which therefore was not a mediator in the relationship between IC and incident disability.

At the ROC analysis performed to determine the predictive value of IC of two-year incident disability, the AUC of IC score was 0.724, and the optimal cutoff value was 77.6. The AUC of the IC score for predicting the two-year incident disability was greater than the AUC of single subdomains. Locomotion had a mean score of 69.4 ± 22.2 and a cutoff of 70.8, with an AUC of 0.652. The cognition subdomain had a mean score of 94.3 ± 13.8 and an AUC of 0.362. The psychology subdomain, with a mean score of 91.1 ± 14.5 and a cutoff of 96.4, showed an AUC of 0.702. Vitality, with a mean score of 92.6 ± 10.5 and a cutoff of 96.4, had an AUC of 0.690. The sensory subdomain, with a mean score of 82.3 ± 17.6 and a cutoff of 35.4, had an AUC of 0.384 (Supplementary Table 3).

Table 2 shows the results of logistic regression models exploring the relationship between IC and the risk of two-year incident disability. High intrinsic capacity (≥ 77.6) was associated with lower likelihood of incident disability (OR 0.13, 95% CI 0.06–0.30, *p* < 0.001). The association remained significant after adjusting for age and sex (OR 0.12, 95% CI 0.05–0.28, *p* < 0.001) as well as in the fully adjusted model (OR 0.11, 95% CI 0.03–0.30, *p* < 0.001).

**Table 2:** Unadjusted and adjusted logistic regression models exploring the association between intrinsic capacity and two-year risk of incident disability

	Unadjusted OR (95% CI)	*p*	Age- and sex-adjusted OR (95% CI)	*p*	Fully adjusted OR (95% CI)	*p*
Intrinsic capacity						
Low (< 77.6)	–		–		–	
High (≥ 77.6)	0.13 (0.06–0.30)	< 0.001	0.12 (0.05–0.28)	< 0.001	0.11 (0.03–0.30)	< 0.001
Age at enrolment			0.96 (0.87–1.06)	0.500	0.95 (0.84–1.06)	0.400
Sex, female			0.81 (0.34–1.95)	0.600	0.66 (0.24–1.85)	0.400
Physically active					1.81 (0.60–6.18)	0.300
Education years					0.82 (0.64–1.02)	0.100
Alcohol abuse					1.01 (0.26–3.51)	0.999
Number of diseases					1.114 (0.81–1.59)	0.500

Abbreviations: CI: confidence interval; HR: hazard ratio

^*^ Covariates included in the logistic regression model were age at enrolment, female sex, regular physical activity, education year, alcohol abuse and the overall number of diseases

In the total sample of 319 participants, 204 (63.9%) had high IC (Table 3). Participants with high IC were younger (*p* < 0.001), more often males (*p* = 0.038), lived alone (*p* < 0.001), were more frequently physically active (*p* < 0.001) and had lower prevalence of recent falls (*p* = 0.001), disability (*p* < 0.001), dementia (*p* < 0.001), Parkinson’s disease (*p* = 0.024), depression (*p* < 0.001), osteoarthritis (*p* = 0.027), multimorbidity (*p* < 0.001), and polypharmacy (*p* = 0.026).

**Table 3:** Characteristics of participants according to intrinsic capacity categories

	Low IC (*n* = 115)	High IC (*n* = 204)	Total sample (*n* = 319)	*p*
*Personal characteristics*				
Age, years	87.2 (5.5)	84.5 (4.1)	85.5 (4.8)	< 0.001
Sex, female	86 (74.8%)	128 (62.7%)	214 (67.1%)	0.038
Education, years	4.9 (1.2)	5.3 (2.0)	5.1 (1.7)	0.080
Living alone	21 (18.3%)	79 (38.7%)	100 (31.3%)	< 0.001
Alcohol abuse	11 (9.6%)	28 (13.7%)	39 (12.2%)	0.362
Active smoking	1 (0.9%)	7 (3.4%)	8 (2.5%)	0.267
Physically active	27 (23.5%)	175 (85.8%)	202 (63.3%)	< 0.001
ADL score	2.6 (2.7)	0.12 (0.62)	1.0 (2.1)	< 0.001
IADL score	4.9 (2.1)	1.5 (1.6)	2.7 (2.5)	< 0.001
ADL disability at enrollment	48 (41.7%)	192 (94.1%)	240 (75.2%)	< 0.001
Recent fall(s)	26 (22.6%)	17 (8.3%)	43 (13.5%)	0.001
*Nutritional status and physical performance*				
BMI, kg/m^2^	24.8 (5.0)	26.3 (4.0)	25.8 (4.4)	0.005
MNA−SF total score	10.9 (1.9)	13.3 (1.1)	12.4 (1.9)	< 0.001
Malnutrition (MNA−SF < 8)	8 (7.0%)	0 (0%)	8 (2.5%)	< 0.001
At risk (MNA−SF 8–11)	53 (46.1%)	15 (7.4%)	68 (21.3%)	< 0.001
Normal (MNA−SF ≥ 12)	54 (47.0%)	189 (92.6%)	243 (76.2%)	< 0.001
SPPB summary score	3.7 (3.0)	8.8 (2.4)	7.0 (3.6)	< 0.001
*Cognition and psychological status*				
CPS score	1.86 (1.85)	0.16 (0.50)	0.77 (1.43)	< 0.001
MDS−DRS score	2.62 (2.41)	0.725 (1.53)	1.41 (2.10)	< 0.001
*Sensory impairment*				
Hearing impairment				
Absent	24 (20.9%)	119 (58.3%)	143 (44.8%)	< 0.001
Mild/moderate	87 (75.7%)	82 (40.2%)	169 (53.0%)	< 0.001
Severe	4 (3.5%)	3 (1.5%)	7 (2.2%)	0.240
Vision impairment				
Absent	42 (36.5%)	145 (71.1%)	187 (58.6%)	< 0.001
Mild/moderate	30 (26.1%)	31 (15.2%)	61 (19.1%)	0.018
Severe	43 (37.4%)	28 (13.7%)	71 (22.3%)	< 0.001
*Intrinsic capacity*				
Total score	64.8 (10.3)	89.3 (6.1)	80.5 (14.2)	< 0.001
Locomotion	30.7 (24.7)	73.7 (19.7)	58.2 (29.9)	< 0.001
Cognition	69.0 (30.8)	97.3 (8.24)	87.1 (23.9)	< 0.001
Psychology	81.3 (17.2)	94.8 (10.9)	89.9 (15.0)	< 0.001
Vitality	77.5 (13.7)	95.1 (7.84)	88.8 (13.3)	< 0.001
Sensory	65.6 (20.5)	85.6 (16.1)	78.4 (20.3)	< 0.001
*Clinical characteristics*				
Coronary artery disease	13 (11.3%)	25 (12.3%)	38 (11.9%)	0.943
Heart failure	10 (8.7%)	6 (2.9%)	16 (5.0%)	0.032
Diabetes mellitus	31 (27.0%)	36 (17.6%)	67 (21.0%)	0.073
COPD	17 (14.8%)	27 (13.2%)	44 (13.8%)	0.829
Dementia	16 (13.9%)	3 (1.5%)	19 (6.0%)	< 0.001
Parkinson’s disease	5 (4.3%)	1 (0.5%)	6 (1.9%)	0.024
Depression	59 (51.3%)	23 (11.3%)	82 (25.7%)	< 0.001
Cancer	7 (6.1%)	7 (3.4%)	14 (4.4%)	0.270
Osteoarthritis	32 (27.8%)	34 (16.7%)	66 (20.7%)	0.027
Number of diseases	2.7 (1.4)	1.8 (1.1)	2.1 (1.3)	< 0.001
Multimorbidity	92 (80.0%)	115 (56.4%)	207 (64.9%)	< 0.001
Number of medications	3.8 (2.2)	2.9 (2.1)	3.2 (2.2)	0.001
Polypharmacy	38 (33.0%)	43 (21.1%)	81 (25.4%)	0.026

Data are reported as means (standard deviations) and absolute numbers (%) for continuous and categorical variables, respectively

Alcohol abuse: ≥500 mL daily of wine or equivalent; multimorbidity: ≥2 chronic diseases; polypharmacy: ≥5 medications

Abbreviations: BMI: body mass index; CPS: cognitive performance scale; COPD: chronic obstructive pulmonary disease; MDS−DRS: Minimum Data Set − Depression Rating Scale; SPPB: short physical performance battery

**Fig. 1:**
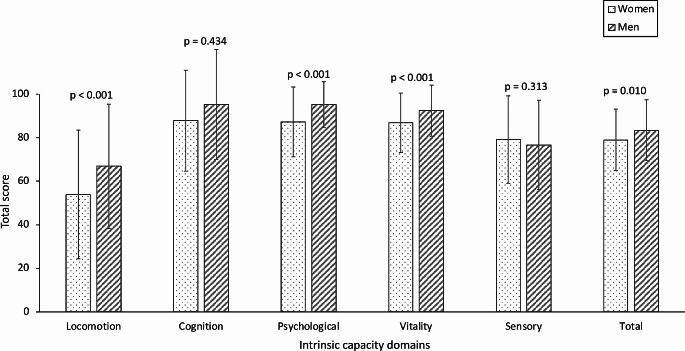
Differences in intrinsic capacity subdomains and total score in men and women

Women exhibited lower mean IC total score (79.0 ± 14.1 vs. 83.4 ± 14.0, *p* = 0.010) and a greater proportion of them were categorized as having low IC (40.2% vs. 27.6%, *p* = 0.038). Men had consistently higher scores in the locomotion (53.9 ± 29.6 vs. 66.9 ± 28.6, *p* < 0.001), psychological (87.3 ± 16.1 vs. 95.3 ± 10.5, *p* < 0.001), and vitality (86.9 ± 13.7 vs. 92.5 ± 11.7, *p* < 0.001) subdomains. No significant differences between men and women were observed regarding cognition or sensory subdomains (Fig. 1). No significant differences in recent falls were reported in women compared to men (15.9% vs. 8.6, *p* = 0.105).

IC and its subdomains exhibited varying degrees of effectiveness in predicting falls, with locomotion showing the highest predictive accuracy among the subdomains. Mean IC score was 80.5 ± 14.2, with an optimal cutoff value of 80.2 and an AUC of 0.701 for predicting recent falls. Among the subdomains, locomotion had a mean of 58.2 ± 29.9 and a cutoff of 37.5, with an AUC of 0.702. Cognition had the lowest predictive ability with a mean of 87.1 ± 23.8, a cutoff of 8.3, and an AUC of 0.414. Psychology, with a mean of 89.9 ± 15.0 and a cutoff of 82.1, had an AUC of 0.568. Vitality and sensory subdomains had means of 88.8 ± 13.3 and 78.4 ± 20.3, cutoffs of 96.4 and 85.4, and AUCs of 0.612 and 0.609, respectively (Supplementary Table 4).

Another mediation analysis was performed to assess the relationship between IC, physical activity, and falls (Supplementary Table 5). In Model A’, the IC effect on falls was −0.043 (*p* < 0.001), indicating a significant negative relationship where higher IC was associated with fewer falls. Model B’ showed that IC positively impacted physical activity, with an effect of 0.152 (*p* < 0.001), suggesting a significant positive association. However, in Model C’, including both IC and physical activity impacting falls, the IC effect on falls decreased to −0.030 and was not statistically significant (*p* = 0.050). This indicates that after accounting for physical activity, IC did not have a significant direct impact on the likelihood of falls, suggesting that the relationship between IC and falls was largely mediated by physical activity. The mediated effect, calculated as the difference between the IC effect in the base model and the IC effect in the complete model, was −0.013. The reduction in the IC effect size from the base model to the complete model suggests that a substantial portion of the effect of IC on falls was mediated by physical activity, which therefore was not included among the potential confounders in the subsequent logistic regression analysis.

The logistic regression models including IC as a continuous variable revealed a significant association between higher IC and lower odds of falls. This association was significant in the unadjusted (OR 0.96, 95% CI 0.94–0.98, *p* < 0.001), the age- and sex-adjusted (OR 0.96, 95% CI 0.94–0.98, *p* < 0.001), and the fully adjusted models (OR 0.96, 95% CI 0.93–0.99, *p* = 0.003). Furthermore, unadjusted logistic regression models exploring the association between IC categories and recent falls showed a strong association between high IC and lower odds of falls (OR 0.31, 95% CI 0.16–0.60, *p* < 0.001). This association remained significant in both the age- and sex-adjusted (OR 0.30, 95% CI 0.15–0.59, *p* < 0.001) and fully adjusted models (OR 0.33, 95% CI 0.16–0.82, *p* = 0.007). Among the subdomains, only locomotion was independently associated with recent falls in the unadjusted (OR 0.98, 95% CI 0.97–0.99, *p* < 0.001), age- and sex-adjusted (OR 0.97, 95% CI 0.96–0.99, *p* < 0.001), and fully adjusted models (OR 0.98, 95% CI 0.96–0.99, *p* < 0.001). Sensory function was significantly associated with recent falls only in the age- and sex-adjusted model (OR 0.98, 95% CI 0.96−0.99, *p* = 0.021). None of the other subdomains of the IC was independently associated with higher odds of recent falls (Table 4).

**Table 4:** Unadjusted and adjusted logistic regression models exploring the association between intrinsic capacity composite score and recent falls and between intrinsic capacity subdomains and recent falls

	Unadjusted OR (95% CI)	*p*	Age- and sex-adjusted OR (95% CI)	*p*	Fully adjusted^*^ OR (95% CI)	*p*
**Intrinsic capacity score**	0.96 (0.94–0.98)	< 0.001	0.96 (0.94–0.98)	< 0.001	0.96 (0.93–0.99)	0.003
**Intrinsic capacity categories**						
Low (< 77.6)	–		–		–	
High (≥ 77.6)	0.31 (0.16–0.60)	< 0.001	0.30 (0.15–0.59)	< 0.001	0.33 (0.16–0.82)	0.007
**Subdomains**						
Locomotion	0.98 (0.97–0.99)	< 0.001	0.97 (0.96–0.99)	< 0.001	0.98 (0.96–0.99)	< 0.001
Cognition	0.99 (0.98–1.00)	0.100	0.99 (0.98–1.00)	0.068	0.99 (0.98–1.00)	0.200
Vitality	0.98 (0.96–1.00)	0.028	0.98 (0.96–1.00)	0.053	0.99 (0.98–1.00)	0.200
Psychology	0.98 (0.97–1.00)	0.100	0.99 (0.97–1.01)	0.200	1.00 (0.98–1.02)	0.999
Sensory	0.99 (0.97–1.00)	0.052	0.98 (0.96–0.99)	0.021	0.98 (0.97–1.00)	0.071

Abbreviations: CI, confidence interval; OR, odds ratio

^*^ Covariates included in the fully adjusted logistic regression model were age at enrolment, female sex, living alone, number of diseases

## Discussion

The present study examined the relationship between IC and recent falls in a well-characterized cohort of individuals aged 80 or older living in the community. IC was measured using a scoring system encompassing five domains: locomotion, cognition, vitality, psychological well-being, and sensory function. To the best of our knowledge, ours is the first study to assess IC using an instrument partially derived from the MDS−HC. Our findings revealed that individuals with higher IC scores were less likely to report a fall in the previous 90 days, with locomotion being an independently associated subdomain.

Despite growing interest in the WHO’s Healthy Ageing framework, both the definition of IC and its translation into clinical practice remain challenging [19, 43]. A recent scoping review of 25 studies published up to 2023 highlighted a significant heterogeneity in the definition of IC compared with the WHO’s proposal and in its subdomains [44]. Notably, 12% of the studies expanded the definition of IC to include six or seven dimensions. There were also significant differences in how the domains of IC were measured and calculated, with varying models such as reflective models, structural-equation modeling (SEM), formative SEM, z-scores, and others [44]. The WHO’s ICOPE guidelines, released in 2017, include evidence-based recommendations for healthcare professionals to prevent, slow, or reverse declines in physical and mental capacities in older adults, alongside a screening tool for IC [45]. However, there are limited data on the diagnostic value of this tool, and research has pointed out its potential drawbacks, including incomplete testing, necessitating a secondary confirmatory instrument [46, 47]. To address these potential limitations, López-Ortiz et al. [18] proposed an alternative instrument to evaluate IC using SPPB, MMSE, MNA, CSDD, and self-reported sensory impairment, yielding a score from 0 to 10. According to the authors, this score may overcome the limitations of the ICOPE screening tool by performing a complete evaluation of the different subdomains from the start of assessment using validated and comprehensive tools [18]. A potential limitation is the lack of detail in specific groups, which might prevent achieving a comprehensive portrayal of diverse, heterogeneous individuals using a construct that spans multiple domains and is inherently complex. An alternative approach, using averaged and rescaled mean scores, may offer more granularity and comparability. A study by Lee et al. [48] involving 1009 Taiwanese older adults found that low IC and greater multimorbidity independently predicted incident disability over a 7-year period, with a 10% reduction in risk for each point increase in IC score. Declines in specific IC subdomains, especially locomotion and psychological health, also predicted disability [48]. Stolz et al. [49] used longitudinal data spanning more than 20 years in 754 older adults aged 70 or older to build an IC score using the POMP method. In their study, the authors obtained an index with fine granularity which declined progressively in later life and was able to predict adverse health outcomes including ADL disability, institutionalization, and need of long-term care placement. These results suggest that monitoring IC could help healthcare providers identify older adults with a low or declining IC who may benefit from preventive interventions years before the onset of dependency [49]. The latter results are particularly significant as the actual importance of IC relies on focusing on longitudinal trajectories rather than on cross-sectional observations [50]. In this regard, a MDS-based instrument to assess IC may be useful to monitor functional decline in older adults over time and across different settings [51]. Nevertheless, none of the existing studies have put forth a universally accepted measuring tool, and the absence of agreement on how to approach and quantify this concept is a significant obstacle to adopting the WHO Healthy Ageing paradigm in both research and clinical practice.

Our study sample included a higher number of women (67.1%), which is reflective of the broader demographic trend where females tend to live longer than males. However, men had better levels of IC and greater scores in the locomotion, psychological and vitality subdomains. This finding is consistent with previous literature, as both Beard et al. [52] and Yu et al. [53] reported lower levels of IC in women. This gender gap is a highly debated topic in geriatric medicine, as females generally age with greater frailty despite their longer lifespan [54, 55]. Sex-related differences were reported in biological and genetical determinants of aging, including differences in the epigenetic clocks [56]. Moreover, socioeconomic inequities further exacerbate the gender gap, particularly in older age. These disparities include unequal access to healthcare, lower income and unpaid labor, less retirement benefits, and limited control over economic resources [55]. From the perspective of IC, this finding underscores the importance of targeted interventions that address gender-specific aging processes. According to our findings, critical areas where women might require more focused support include psychological wellbeing and factors influencing physical performance and metabolism, including nutritional status and access to physical activity. However, further studies are required to identify sex-specific factors influencing functional reserves across the lifespan.

Another interesting finding of our study is that physical activity was an independent contributor to the onset of incident disability and did not mediate the relationship between IC and incident disability among participants without pre-existing ADL impairment. Conversely, physical activity was found to act as a significant mediator in the relationship between IC and falls in the total study sample. Additionally, the prevalence of physical activity was significantly greater in individuals with higher IC than in participants with lower IC (85.8% vs. 23.5%, *p* < 0.001). This finding suggests that IC may facilitate the ability to engage in physical activity, highlighting the interdependence between overall health and physical activity. Higher IC, which encompasses various health dimensions appears to enable individuals to maintain a more active lifestyle. As reported previously by Mangani et al. [24] using data from the same cohort, this active lifestyle, in turn, is crucial for preventing falls, indicating a feedback loop where maintaining high IC supports physical activity which then helps preserve IC by preventing fall-related injuries [57]. These insights underscore the importance of holistic health interventions aimed at enhancing IC to promote sustained physical activity and prevent adverse outcomes, including falls, in older adults.

Regarding the relationship between decreased IC and risk of falls, our findings are consistent with existing evidence. According to Charles et al. [58], certain domains of the IC, namely a higher balance performance (HR 0.87, 95% CI 0.79–0.96) and better nutritional status (hazard ratio [HR] 0.96, 95% CI 0.93–0.98), were associated with a decreased risk of falling during a three-year follow-up. However, it is important to note that Charles et al. [58] did not find an association between overall IC and the risk of recurrent falls. This highlights that, while specific IC domains may reduce the risk of initial falls, their impact on preventing recurrent falls might be different. Liu et al. [59] investigated the predictive value of IC and a modified frailty phenotype for adverse outcomes in a cohort of 212 octogenarians. They reported a significant association between IC, particularly in certain subdomains, and higher odds of falling. Relevant subdomains were low performance on the chair stand (OR 3.102, 95% CI 1.406–6.845), weight loss (OR 6.282, 95% CI 1.203–32.802), and reduced interest in doing things (OR 2.708, 95% CI 1.183–6.202). When compared using ROC analysis, IC showed better predictive performance for falls than frailty based on the AUC [59]. While both Charles et al. [58] and Liu et al. [59], consistent with our findings, demonstrated an association between the risk of falls and subdomains related to locomotion or physical performance, a separate study by Lu et al. [60] did not report an association between limited mobility and the risk of recurrent falls (≥ 1 during the previous year) (OR 0.53, 95% CI 0.17–1.61). In contrast, an association was found between recurrent falls and visual impairment (OR 2.85, 95% CI 1.12–7.21). A study using data from 975 adults aged 20–102 years living in the Toulouse area (France) and enrolled in the INSPIRE-T cohort displayed the distribution of IC across the life course and proposed reference centiles for different ages [61]. Accordingly, individuals below the 10th centile of IC had significantly greater odds of reporting falls in the previous three months (OR 31.4, 95% CI 4.1–238.8, *p* < 0.001). Additionally, individuals between the 26th and 75th centiles of IC had a higher prevalence of recent falls compared with those above the 90th centile [61]. Meng et al. [62] investigated the relationship between different IC subdomains and age-related outcomes in a sample of 1728 older adults from the Taiwan Longitudinal Study on Ageing (TLSA). Their results showed that an IC pattern characterized by physio-cognitive decline and depression was associated with a higher risk of falls (OR 1.62, 95% CI 1.18–2.23, *p* < 0.01) [62]. Shen et al. [63] found an increased risk of falls associated with declining IC composite scores in a sample of 703 old inpatients. The OR for the composite score was 0.64 (95% CI 0.57–0.72). Noticeably, specific subdomains were linked to an increased risk of falls, including cognition (OR 0.38, 95% CI 0.28–0.53, *p* < 0.001), vitality (OR 0.52, 95% CI 0.40–0.66, *p* < 0.001), locomotion (OR 0.26, 95% CI 0.19–0.38, *p* < 0.001), and psychology (OR 0.70, 95% CI 0.47–0.94, *p* = 0.022) [63]. Finally, Muneera et al. [64] demonstrated an association between low IC and multiple falls (OR 0.73, 95% CI 0.58–0.96) and fall-related injury (OR 0.78, 95% CI 0.61–0.99) in a sample of 24,136 older adults from the Longitudinal Aging Study in India (LASI).

The association between falls and IC is not unexpected. Falls in older adults are influenced by various factors, including external determinants such as inadequate illumination, uneven surfaces, tripping hazards, and slippery floors, as well as personal risk factors. This suggests that preserving and improving an individual’s IC is a primary strategy for preventing falls in older adults [65]. The World Guidelines for Falls Prevention and Management address several modifiable risk factors for falls, such as poor balance, gait problems, sarcopenia, fear of falling, impaired hearing and vision, cognitive impairment, co-existing conditions including depression, and poor nutritional status [66]. It should be noted that most personal factors reflect the five domains of IC [17]. While current research has demonstrated a clear association between decreased IC and adverse health outcomes [44, 67], the next and more relevant steps involves understanding the trajectories and patterns of IC, how they are negatively influenced across the lifetime, and what actions can increase and maintain functional ability in older adults. In this context, an MDS-based instrument to assess IC may be useful for monitoring the decline of functional reserves in older adults over time and across different settings [51].

Although reporting intriguing findings, our study has limitations that deserve acknowledgment. Because participants were selected from individuals who had already reached 80 years of age at enrolment, selection of high IC cannot be ruled out. Differences in health literacy or access to healthcare services might have existed between groups. However, since participants lived in a well-defined geographic area and education levels did not differ between groups, it is unlikely that individuals with higher IC received better care than those with lower IC. Additionally, since ilSIRENTE only included adults 80 years or older, our results may not be applicable to younger age groups. Likewise, they may not be generalizable to non-Caucasian ethnicities. Another notable limitation is that the enrolled population appears to be fitter than the typical cohort accessing health services. The average IC scores and physical performance metrics indicate a relatively high level of functioning among our participants. This selection bias might be due the community-dwelling nature of the sample and the recruitment methods. Additionally, the inclusion of individuals over 80 years of age may have led to a natural selection process, excluding less fit individuals who may have died earlier. The higher-than-average fitness levels could have implications for the generalizability of our findings, suggesting that the observed associations and interventions might be more applicable to older adults who are relatively healthy and active. Conversely, it highlights the potential for even greater benefits in more frail populations who might incur more severe consequences from a further decline in functional abilities as well as more pronounced improvements in IC with appropriate interventions. Furthermore, the size of the total sample (*n* = 319) and the lack of follow-up information on the subdomains of IC prevented us from analyzing IC trajectories using either a partially or fully MDS-derived IC composite score. Lastly, the possibility of unmeasured factors influencing the study results cannot be ruled out.

## Conclusions

Findings from the present study indicate that an MDS-derived IC score is independently associated with the risk of falls in very old adults living in the community, particularly highlighting the locomotion subdomain. This finding, albeit preliminary, supports the use MDS to measure IC and monitor its decline in older adults over time and across different settings.

### Electronic supplementary material

Below is the link to the electronic supplementary material.

Supplementary Material 1

## Data Availability

The data that support the findings of this study are available on request from the corresponding author. The data are not publicly available due to their containing information that could compromise the privacy of the research participants.
