# Supplementary material for: Intrinsic capacity and recent falls in adults 80 years and older living in the community: results from the ilSIRENTE Study
Source: Aging Clin Exp Res. 2024 Aug 10;36(1):169. doi: 10.1007/s40520-024-02822-7 (PMC11316723; doi:10.1007/s40520-024-02822-7)
Supplement: Supplementary file 1 — Supplementary Material 1 [file 40520_2024_2822_MOESM1_ESM.docx]

**Supplementary table 1.** Characteristics of participants with and withouth history of recent falls (< 90 days).

|  | **No recent falls**  **(n=276)** | **Recent falls**  **(n=43)** | **Total sample (n=319)** | **p** |
| --- | --- | --- | --- | --- |
| *Personal characteristics* |  |  |  |  |
| Age, years | 85.5 (4.90) | 85.3 (4.41) | 85.5 (4.83) | 0.828 |
| Sex, female | 180 (65.2%) | 34 (79.1%) | 214 (67.1%) | 0.105 |
| Education, years | 5.14 (1.78) | 5.14 (1.52) | 5.14 (1.74) | 0.994 |
| Living alone | 86 (31.2%) | 14 (32.6%) | 100 (31.3%) | 0.999 |
| Alcohol abuse | 32 (11.6%) | 7 (16.3%) | 39 (12.2%) | 0.534 |
| Active smoking | 8 (2.9%) | 0 (0%) | 8 (2.5%) | 0.604 |
| Physically active | 185 (67.0%) | 17 (39.5%) | 202 (63.3%) | 0.001 |
| ADL score | 0.86 (1.93) | 2.09 (2.55) | 1.03 (2.06) | 0.004 |
| IADL score | 2.56 (2.42) | 3.70 (2.46) | 2.71 (2.45) | 0.007 |
| ADL disability at enrollment | 217 (78.6%) | 23 (53.5%) | 240 (75.2%) | 0.001 |
| *Nutritional status and physical performance* | | | | |
| BMI, kg/m^2^ | 25.7 (4.24) | 26.4 (5.53) | 25.8 (4.43) | 0.394 |
| MNA−SF total score | 12.5 (1.82) | 11.8 (2.06) | 12.4 (1.87) | 0.044 |
| Malnutrition (MNA−SF <8) | 6 (2.2%) | 2 (4.7%) | 8 (2.5%) | 0.658 |
| At risk (MNA−SF 8–11) | 58 (21.0%) | 10 (23.3%) | 68 (21.3%) | 0.814 |
| Normal (MNA−SF ≥12) | 212 (76.8%) | 31 (72.1%) | 243 (76.2%) | 0.629 |
| SPPB summary score | 7.33 (3.47) | 4.72 (3.52) | 6.98 (3.59) | <0.001 |
| *Cognition and psychological status* |  |  |  |  |
| CPS score | 0.721 (1.41) | 1.12 (1.56) | 0.774 (1.43) | 0.124 |
| MDS−DRS score | 1.33 (2.06) | 1.91 (2.29) | 1.41 (2.10) | 0.125 |
| *Sensory impairment* |  |  |  |  |
| Hearing impairment |  |  |  |  |
| Absent | 131 (47.5%) | 12 (27.9%) | 143 (44.8%) | 0.025 |
| Mild/moderate | 139 (50.4%) | 30 (69.8%) | 169 (53.0%) | 0.027 |
| Severe | 6 (2.2%) | 1 (2.3%) | 7 (2.2%) | 0.999 |
| Vision impairment |  |  |  |  |
| Absent | 168 (60.9%) | 19 (44.2%) | 187 (58.6%) | 0.057 |
| Mild/moderate | 50 (18.1%) | 11 (25.6%) | 61 (19.1%) | 0.304 |
| Severe | 58 (21.0%) | 13 (30.2%) | 71 (22.3%) | 0.248 |
| *Intrinsic capacity* |  |  |  |  |
| Total score | 81.7 (14.0) | 72.9 (12.9) | 80.5 (14.2) | <0.001 |
| Locomotion | 61.1 (28.9) | 39.3 (29.3) | 58.2 (29.9) | <0.001 |
| Cognition | 88.0 (23.4) | 81.4 (26.0) | 87.1 (23.9) | 0.124 |
| Psychology | 90.5 (14.7) | 86.4 (16.3) | 89.9 (15.0) | 0.125 |
| Vitality | 89.4 (13.0) | 84.6 (14.7) | 88.8 (13.3) | 0.044 |
| Sensory | 79.3 (20.5) | 72.8 (18.1) | 78.4 (20.3) | 0.035 |
| *Clinical characteristics* |  |  |  |  |
| Coronary artery disease | 34 (12.3%) | 4 (9.3%) | 38 (11.9%) | 0.753 |
| Heart failure | 13 (4.7%) | 3 (7.0%) | 16 (5.0%) | 0.797 |
| Diabetes mellitus | 53 (19.2%) | 14 (32.6%) | 67 (21.0%) | 0.072 |
| COPD | 40 (14.5%) | 4 (9.3%) | 44 (13.8%) | 0.496 |
| Dementia | 16 (5.8%) | 3 (7.0%) | 19 (6.0%) | 0.999 |
| Parkinson’s disease | 6 (2.2%) | 0 (0%) | 6 (1.9%) | 0.709 |
| Depression | 65 (23.6%) | 17 (39.5%) | 82 (25.7%) | 0.041 |
| Cancer | 12 (4.3%) | 2 (4.7%) | 14 (4.4%) | 0.999 |
| Osteoarthritis | 54 (19.6%) | 12 (27.9%) | 66 (20.7%) | 0.292 |
| Number of diseases | 2.08 (1.26) | 2.56 (1.45) | 2.14 (1.30) | 0.046 |
| Multimorbidity | 174 (63.0%) | 33 (76.7%) | 207 (64.9%) | 0.114 |
| Number of medications | 3.12 (2.17) | 3.79 (2.25) | 3.21 (2.19) | 0.073 |
| Polypharmacy | 69 (25.0%) | 12 (27.9%) | 81 (25.4%) | 0.827 |

Data are reported as means (standard deviations) and absolute numbers (%) for continuous and categorical variables, respectively.

Alcohol abuse: ≥500 mL daily of wine or equivalent; multimorbidity: ≥2 chronic diseases; polypharmacy: ≥5 medications.

Abbreviations: BMI: body mass index; CPS: cognitive performance scale; COPD: chronic obstructive pulmonary disease; MDS−DRS: Minimum Data Set − Depression Rating Scale; SPPB: short physical performance battery.

**Supplementary table 2.** Mediation analysis summary for intrinsic capacity, physical activity, and incident disability.

| **Model** | **Effect** | **p** |
| --- | --- | --- |
| Model A (Intrinsic capacity impacts incident disability) | -0.014 | <0.001 |
| Model B (Intrinsic capacity impacts physical activity) | 0.045 | 0.003 |
| Model C (Intrinsic capacity and physical activity impact falls) | -0.016 | <0.001 |
| Mediated effect | 0.004 |  |

**Supplementary table 3.** Means and optimal cut-off values of intrinsic capacity and its subdomains for predicting incident disability determined by receiver operating characteristic analysis.

|  | **Mean (SD)** | **Optimal cut-off** | **AUC** |
| --- | --- | --- | --- |
| Intrinsic capacity | 85.9 (9.6) | 77.6 | 0.724 |
| *Subdomains* |  |  |  |
| Locomotion | 69.4 (22.2) | 70.8 | 0.652 |
| Cognition | 94.3 (13.8) | – | 0.362 |
| Psychology | 91.1 (14.5) | 96.4 | 0.702 |
| Vitality | 92.6 (10.5) | 96.4 | 0.690 |
| Sensory | 82.3 (17.6) | 35.4 | 0.384 |

Abbreviations: AUC: area under the curve; SD: standard deviation.

**Supplementary table 4.** Means and optimal cut-off values of intrinsic capacity and its subdomains for predicting falls determined by receiver operating characteristic analysis.

|  | **Mean (SD)** | **Optimal cut-off** | **AUC** |
| --- | --- | --- | --- |
| Intrinsic capacity | 80.5 (14.2) | 80.2 | 0.701 |
| *Subdomains* |  |  |  |
| Locomotion | 58.2 (29.9) | 37.5 | 0.702 |
| Cognition | 87.1 (23.8) | 8.3 | 0.414 |
| Psychology | 89.9 (15.0) | 82.1 | 0.568 |
| Vitality | 88.8 (13.3) | 96.4 | 0.612 |
| Sensory | 78.4 (20.3) | 85.4 | 0.609 |

Abbreviations: AUC: area under the curve; SD: standard deviation.

**Supplementary table 5.** Mediation analysis summary for physical activity, intrinsic capacity, and falls.

| **Model** | **Intrinsic capacity effect** | **p** |
| --- | --- | --- |
| Model A’ (Intrinsic capacity impacts falls) | -0.043 | <0.001 |
| Model B’ (Intrinsic capacity impacts physical activity) | 0.152 | <0.001 |
| Model C’ (Intrinsic capacity and physical activity impact falls) | -0.030 | 0.050 |
| Mediated effect | -0.013 |  |
